# Demographic variation in charitable giving and helping across 22 countries in the Global Flourishing Study

**DOI:** 10.1038/s41598-025-96009-3

**Published:** 2025-04-30

**Authors:** Julia S. Nakamura, Dorota Węziak-Białowolska, Robert D. Woodberry, Laura D. Kubzansky, Koichiro Shiba, R. Noah Padgett, Byron R. Johnson, Tyler J. VanderWeele

**Affiliations:** 1 Department of Psychology, University of British Columbia, 2136 West Mall, Vancouver, BC V6T 1Z4 Canada; 2 Department of Quantitative Methods and Information Technology, Kozminski University, Warsaw, Poland; 3 Human Flourishing Program, Institute for Quantitative Social Science, Harvard University, Cambridge, USA; 4 Institute for Studies of Religion, Baylor University, Waco, USA; 5 Department of Social and Behavioral Sciences, Harvard T.H. Chan School of Public Health, Boston, USA; 6 School of Public Health, Boston University, Boston, USA; 7 Department of Biostatistics, Harvard T.H. Chan School of Public Health, Boston, USA; 8 Department of Epidemiology, Harvard T.H. Chan School of Public Health, Boston, USA

**Keywords:** Global Flourishing Study, Prosocial behavior, Charitable giving, Helping strangers, Demographics, Cross-national, Public health, Human behaviour

## Abstract

Prosocial behaviors play a vital role in promoting individual and societal well-being. Charitable giving and helping strangers are two important expressions of prosociality; yet we know little about how these behaviors differ across sociodemographic indicators cross-nationally. Using data from the Global Flourishing Study, a diverse and international sample of 202,898 individuals across 22 countries, we examined distributions of charitable giving and helping (binary variables, yes/no) across nine demographic factors (age, gender, marital status, employment status, religious service attendance, education, immigration status, race and ethnicity, and religious affiliation) and culturally diverse countries. Unadjusted proportions of charitable giving and helping in the past month varied substantially between countries for charitable giving (from 0.10 [Japan] to 0.79 [Indonesia]) and helping (from 0.11 [Japan] to 0.83 [Nigeria]). Random effects meta-analyses confirmed that rates of charitable giving and helping differed among some demographic groups (e.g., more charitable giving with older age, less helping with older age, increased charitable giving and helping with more education) and that rates of charitable giving and helping across all demographic factors differed between countries. Better understanding how various sociodemographic factors may be associated with prosocial behaviors, and how these associations differ internationally, may help to inform interventions designed to enhance prosociality around the world.

**Supplementary Information:**

The online version contains supplementary material available at 10.1038/s41598-025-96009-3.

## Introduction

In one afternoon at a local coffee shop, suppose you observed someone holding the door open for the next patron, another person buying a coffee for the person next in line, or someone else returning a forgotten cell phone. These voluntary acts intended to benefit others are examples of prosocial behaviors^1^. Prosocial behaviors play a vital role in shaping our communities and promoting individual well-being^2^. Charitable giving (i.e., donating money to a charity) and helping strangers (i.e., helping someone you do not know) are two common ways people express their prosociality (amongst others, such as cooperation) and are important components of voluntary action.

Charitable giving and helping strangers are common worldwide and are associated with better societal and individual well-being. In 2021, 62% of adults over age 15 reported that they helped a stranger (~ 3.5 billion people) and 35% reported having donated to charity (nearly 2 billion people) in the past month; in fact, in this same year, reported rates of these two prosocial behaviors reached their highest recorded levels globally since 2009, although rates clearly vary between countries^4^. Charitable giving and helping also contribute billions to the global economy and undergird civil society^5^. Beyond such societal benefits, they also appear to provide health and well-being benefits. Indeed, research demonstrates associations between charitable giving and helping others and better health and well-being outcomes. For example, charitable giving has been associated with increased happiness^6,7^, and helping behaviors have been associated with reduced risk of mortality and stroke, increased happiness, higher purpose in life, and other improved health and well-being outcomes^8,9^. Some forms of prosociality (e.g., small acts of kindness to a stranger) may be more likely to enhance community-level factors that also promote health (e.g., community cohesion), making prosociality a promising public health target for enhancing societal health and well-being^2^.

As we continue to examine when and how charitable giving and helping may contribute to the well-being of individuals and societies, it is important to understand how participation in these activities differs across countries and sociodemographic groups. It is important to evaluate demographic groups specifically for several reasons, including 1) policy implications (i.e., many interventions, funding decisions, and policies are made according to demographic data rather than psychological profiles, potentially making demographic analysis more actionable in informing social programs and charitable initiatives); 2) their consistency between countries, which may be higher than for psychological factors; and 3) because they serve as markers of (and reminders to consider) systemic and structural factors in society that may constrain or promote prosocial behaviors (e.g., demographics help identify broad patterns of inequality, privilege, and structural constraints that influence prosocial behaviors), amongst other reasons. Charitable giving and helping are conceptually and practically different ways of engaging in prosociality and may be distributed differentially across social groups and countries based on social and cultural norms. It is of practical importance for organizations, governments, and public health officials to know who gives and helps. If some demographic groups have lower rates of charitable giving and helping, interventionists may consider barriers and facilitators to participation for these groups to ensure equitable promotion of individual and national well-being. Better understanding where charitable giving and helping are (or are not) common internationally, with further research, may inform interventions designed to enhance global prosociality. Of course, if individuals with lower education and income are less likely to engage in charitable giving but more likely to help others directly, the approach would not be to promote costly giving that is unrealistic for these individuals. Rather, interventions could focus on strengthening informal helping networks and removing barriers to direct prosocial engagement, rather than emphasizing monetary donations. By identifying sociodemographic differences in prosocial behaviors, policymakers and organizations can aim to tailor their outreach strategies to ensure that all individuals, regardless of socioeconomic status, have meaningful opportunities to contribute to their communities in ways that are feasible, culturally accessible, and ideally health promoting. Below, we briefly review prior literature examining how sociodemographic factors may be associated with prosocial behaviors.

While prior literature has documented individual differences in the likelihood and extent of charitable giving and helping, associations between several demographic factors and rates of charitable giving and helping have not been consistent in prior research^10,11^. Not surprisingly, aging is associated with a variety of changes that may influence prosocial engagement. For example, several studies (mostly in the United States [US] and other Western, Educated, Industrialized, Rich, and Democratic [WEIRD]^12^countries and a few in other countries [e.g., Taiwan, Japan]) have found that older adults typically donate more money than younger age groups^10^, potentially due to increased financial stability, higher levels of disposable income, and a greater sense of social responsibility (though in some countries, depending on pension systems, financial conditions are more unfavorable for older adults). Helping may likewise differ across age groups; for example, having school-aged children in midlife may increase informal helping due to higher engagement in networks of mutual assistance, and helping may decrease with age-related health problems^11^.

Regarding gender differences in charitable giving, findings are mixed^13^: while women may be more motivated to give and help, gender differences in social capital (e.g., social networks) and resources, in which men are typically advantaged, may result in rates of giving and helping looking similar across men and women^14^. When more socioeconomic variables (e.g., income, education) are included in models examining charitable giving, the reported gender differences in charitable giving become smaller^13,15^. Regarding helping, some work has found that women engage in more helping than men^11,16^, while other work has shown the reverse; however the extent to which these findings are an artefact of measurement error remains unclear^17^.

Ethnic minority status has been associated with decreased charitable giving in some WEIRD countries^15^; however, other studies do not find differences after accounting for other sociodemographic factors such as income, education, and immigration status^15,18^. Some work in the US and Australia has found that informal helping (i.e., “unpaid volunteering not coordinated by an organization or institution”) directed toward helping people outside of one’s household (e.g., babysitting, cooking meals, etc.)^11^ is more common among ethnic minorities, though research is limited on associations between ethnic minority status and rates of helping strangers.

Most studies find that being married is associated with increased rates of charitable giving^13,15^, potentially due to the shared financial resources (e.g., more wealth)^15^and larger and more diverse social networks through which they may be solicited for contributions within married couples. Findings for whether or not being married is associated with rates of helping are mixed^19^.

Stronger data are available showing associations between markers of socioeconomic status (SES) and prosocial behaviors. Generally, higher education, greater income and wealth, home ownership, better subjective financial position, and employment have been associated with more charitable giving, although most of this work has been conducted in WEIRD countries^10,15^. Findings are less consistent with regard to the relationship between different markers of SES and helping behaviors^11^: some studies find that people with low income and education are more likely to engage in informal helping, while other research observes a positive relationship between occupational prestige and more education and informal helping^11,20^.

Many religious organizations promote charitable giving and helping behaviors, so it would not be surprising to find that prosocial behaviours have been associated with religious service attendance and membership in religious organizations. Indeed, membership in a religious organization and frequency of religious service attendance have been associated with (a) increased charitable giving and higher amounts donated in multiple studies in mostly WEIRD countries^10,15^and (b) helping strangers in a study of 126 countries^21^. There also seems to be some variation in the amounts of charitable giving and helping strangers across different religious traditions, although the reasons for it are less well understood^10,22,23^.

Findings for immigration status yield mixed results. For example, some studies (in the US) have found that immigrant status is associated with reduced charitable giving (e.g., immigrants may prioritize giving informally through immigrant remittances [i.e., informal gifts of money and goods to relatives, friends, and others in need in the immigrant’s country of origin])^15^. Other work has found that immigrants are just as (or more) likely than native-born individuals to donate (especially with more time after immigration) and give more when donating^24,25^. When considering helping behaviors, there is a shortage of empirical research and findings tend to be inconclusive. For example, in Canada, residential areas with more immigrants showed greater helping, while in the US, residential areas with more immigrants may be less likely to help^26^.

Because prosociality is important for stable societies, it is not surprising that prosociality and altruism are found cross-culturally^27,28^. Yet, cultural comparisons reveal substantial diversity in terms of cooperation in adulthood and in other prosocial norms^29–33^. Recent comparisons from the 2023 World Giving Index^34^showed large differences across nations in rates of charitable giving and helping. The countries that had the highest rates of charitable giving were Myanmar, Indonesia, the United Kingdom (UK), Ukraine, Malta, and Sweden (all with rates of 65% or greater), and those with the lowest rates were Egypt, Lesotho, Georgia, Afghanistan, Tunisia, Greece, Yemen, and Morocco (all with rates less than 10%). The countries where people were most likely to help strangers were Jamaica, Liberia, Libya, Nigeria, Kuwait, Ukraine, Senegal, Kenya, the US, and Sierra Leone (all with rates of 76% or greater), while Cambodia, Poland, and Japan had lower rates of helping (all with rates of 22% or lower; cf^34^).

Various contextual factors may explain why we see differences in prosocial behaviors across nations, including differences in: social trust; religious and moral values; rates of technological and globalization change (which have expanded opportunities for charitable giving in some settings); government and institutional context (e.g., tax incentives for charitable donations, government support or regulation of nonprofit organizations that may crowd out or encourage private contributions by giving nonprofit organizations greater resources for fundraising); philanthropic traditions (e.g., notions of social responsibility); cultural variations in perceptions of risk and safety; economic development and stability/growth; industrialization; level of religious liberty; type, size, and scope of the nonprofit sector; historical factors; population changes; international giving; and more^3,4,10,35–37^.

While these existing studies have made meaningful contributions to our understanding of charitable giving and helping in adulthood cross-nationally, there are several limitations that inhibit a more robust understanding of the nature of these associations. First, though many studies assess rates of charitable giving and helping globally^4,38,34^, few studies also assess sociodemographic differences in rates of charitable giving and helping strangers cross-nationally. Assessing sociodemographic factors provides additional insights into who engages in charitable giving and helping within countries and how this differs between countries, which may provide more accurate assessments for policymakers as well as interventionists who aim to enhance global prosociality. Second, acknowledging the impact of recent global events (e.g., the Covid-19 pandemic which likely resulted in changes relevant to prosocial engagement such as an increased need for charitable giving and physical distancing policies which likely reduced contact with strangers^39^), it is essential to update our understanding of these behaviors to reflect current worldwide demographic trends in charitable giving and helping. Though recent work has examined worldwide trends^34^, these reports do not assess demographic factors. As these two prosocial behaviors (along with volunteering) are the most commonly assessed in relation to health and well-being and have been associated with better health and well-being, it is important that we understand their global patterns. Third, it is especially important to study charitable giving and helping at the same time. Time and money may provide different paths for expressing prosociality with some behaviors being more or less accessible to individuals depending on their personal and social context. For example, for poorer individuals and countries, time may be more available and money less so. Whereas, for wealthier individuals and countries, money may be more available and time may be more constrained. Thus, some countries may rank higher on giving money, but lower on giving time (or vice versa).

In the present study, we examine the distributions of charitable giving and helping across countries and sociodemographic groups, using data from a diverse and international sample of 202,898 individuals across 22 countries. We use an exploratory approach to identify demographic patterns of charitable giving and helping in order to inform further investigation and potential subsequent theory generation in future research. First, we assess how the proportion of participants who engaged in charitable giving and helping a stranger in the past month (in separate models) varies across countries. Second, we assess how the proportion of participants who engaged in charitable giving and helping a stranger in the past month varies across different demographic categories, including age, gender, marital status, employment status, education, religious service attendance, immigration status, race and ethnicity, and religious affiliation in each country. We report the sociodemographic distributions of charitable giving and helping in each country and also globally via meta-analysis. We explore how rates of charitable giving and helping are patterned across a diverse set of countries with nationally representative samples^40^, how these rates vary across a more comprehensive set of demographic variables than has been incorporated to date in a comparative setting, and how countries differ in sociodemographic patterns of charitable giving and helping. Based on mixed findings in prior research, we have taken an exploratory approach and do not have precise, directional hypotheses. As such, we anticipate that charitable giving and helping will exhibit variations across different demographic categories, and that these differences across demographic categories will themselves vary by country.

## Methods

The description of the methods below have been adapted from other materials^41^. Further methodological detail is available elsewhere^40,42–47,48^.

### Study population

The Global Flourishing Study (GFS) is a study of 202,898 participants from 22 geographically and culturally diverse countries, with nationally representative sampling within each country, concerning the distribution of determinants of well-being. Wave 1 of the data included the following countries and territories: Argentina, Australia, Brazil, Egypt, Germany, Hong Kong, India, Indonesia, Israel, Japan, Kenya, Mexico, Nigeria, the Philippines, Poland, South Africa, Spain, Sweden, Tanzania, Turkey, the UK, and the US. The countries were selected to (a) maximize coverage of the world’s population, (b) ensure geographic, cultural, and religious diversity, and (c) prioritize feasibility and leverage existing data collection infrastructure. Data collection was carried out by Gallup Inc. Data for Wave 1 were collected principally during 2023, with some countries beginning data collection in 2022 and exact dates varying by country^44^. Four additional waves of panel data on the participants will be collected annually from 2024 to 2027. The precise sampling design to ensure nationally representative samples varied by country and further details are available elsewhere^44^. Survey items were all obtained via self-report (in face-to-face, telephone, or online surveys) and included aspects of well-being such as happiness, health, meaning, character, relationships, and financial stability^49^, along with other demographic, social, economic, political, religious, personality, childhood, community, health, and well-being variables. The data are publicly available through the Center for Open Science (COS; https://www.cos.io/gfs). During the translation process, Gallup adhered to the TRAPD model (translation, review, adjudication, pretesting, and documentation) for cross-cultural survey research (ccsg.isr.umich.edu/chapters/translation/overview). Ethical approval was granted by the institutional review boards at Baylor University (IRB Reference #: 1841317) and Gallup (IRB Reference #: 2021-11-02). Gallup is a multi-national corporation and its IRB covers all countries included in the GFS. All participants provided informed consent. The research conformed to the principles of the Helsinki Declaration.

### Measures

#### Demographics variables

Continuous age was classified as 18–24, 25–29, 30–39, 40–49, 50–59, 60–69, 70–79, and 80 or older. Gender was assessed as male, female, or ‘other’. Marital status was assessed as single/never married, married, separated, divorced, widowed, and domestic partner. Employment was assessed as employed, self-employed, retired, student, homemaker, unemployed and searching, and ‘other’. Education was assessed as up to 8 years, 9–15 years, and 16 + years. Religious service attendance, asked as ‘How often do you attend religious services?’, was assessed as more than once/week, once/week, one-to-three times/month, a few times/year, or never. Immigration status was dichotomously assessed with one item: “Were you born in this country, or not?” Response options included ‘born in this country’ or ‘born in another country.’ Religious tradition/affiliation was characterized using the categories of Christianity, Islam, Hinduism, Buddhism, Judaism, Sikhism, Baha’i, Jainism, Shinto, Taoism, Confucianism, Primal/Animist/Folk religion, Spiritism, African-Derived, some other religion, or no religion/atheist/agnostic; precise response categories varied by country^45^. Racial/ethnic identity was assessed in some but not all countries, with response categories varying by country. For additional details on the assessments, see the COS GFS codebook or other materials (cf^40^).

*Outcome Variables.* Charitable giving was assessed with a single item, which asked, “In the past month, have you donated money to a charity?” Helping strangers was assessed with a single item, which asked, “In the past month, have you helped a stranger or someone you didn’t know who needed help?” Response options were binary yes/no for both items.

### Statistical analysis

We estimated descriptive statistics for the full sample, weighted to be nationally representative within each country, for each of the demographic variables. Nationally representative proportions for charitable giving and helping were estimated separately for each country and are presented in order from highest to lowest along with 95% confidence intervals and standard deviations. Variation in proportions for charitable giving and helping across demographic categories were estimated, with all analyses initially conducted separately within country (see Supplementary Material). The primary outcome was global proportions of charitable giving or helping behaviors obtained by conducting random effects meta-analyses of country-specific proportions of charitable giving or helping behavior in each specific demographic category^50,51^ along with 95% confidence intervals, standard errors, lower and upper limits of a 95% prediction interval across countries, heterogeneity (τ), and I^2^for evidence concerning variation within a particular demographic variable across countries^52^. Forest plots of estimates to help illustrate the heterogeneity of the proportions of charitable giving and helping by country are available in the online supplement. All meta-analyses were conducted in **R**^53^using the metafor package^54^. Within each country, we conducted a global test of variation of charitable giving and helping across levels of each particular demographic variable, and a pooled *p*-value^55^ across countries was computed to test whether there was evidence of an effect in any country. Bonferroni corrected *p*-value thresholds are provided based on the number of demographic variables considered (i.e., *p*< .007)^56,57^. Religious affiliation/tradition and race/ethnicity were used when available, and results are presented in the online supplement of country-specific results and not meta-analyzed because the frequency of observed responses led to convergence issues (i.e., religious affiliation/tradition) or the categories themselves differed by country (i.e., race/ethnic identity). As a supplementary analysis, we conducted population weighted meta-analyses. The population weighted meta-analysis effectively treats each person in the 22 countries equally, rather than treating each of the 22 countries equally as random effects meta-analyses do. All analyses were pre-registered with COS prior to data access (https://doi.org/10.17605/OSF.IO/E9URJ); all code to reproduce analyses are openly available in an online repository^43^.

### Missing data

We imputed missing data on all variables using multivariate imputation by chained equations and five imputed datasets were used^58–61^. To account for variation in the assessment of certain variables across countries (e.g., religious affiliation/tradition and race/ethnicity), we conducted the imputation process separately in each country. This within-country imputation approach ensured that the imputation models accurately reflected country-specific contexts and assessment methods. Sampling weights were included in the imputation model to account for missingness related to probability of study inclusion.

### **Accounting for** complex sampling design

The GFS used different sampling schemes across countries based on availability of existing panels and recruitment needs^44^. All analyses accounted for the complex survey design components by including weights, primary sampling units, and strata. Additional methodological details, including accounting for the complex sampling design, are provided elsewhere^46,47^.

## Results

Table 1 provides nationally representative descriptive statistics on demographic characteristics of the overall sample (all 22 countries combined). The sample was mostly evenly distributed across age groups (apart from participants aged over 80) and gender (apart from ‘other’ gender identities). Moreover, 53% of participants were married, 39% were employed for an employer, 62% attended religious services, 57% had 9–15 years of education, and 94% were native to their country. The Supplementary Material provides nationally representative descriptive statistics across demographic categories by country (see Tables S1a-S22a). Meta-analyzing across these 22 countries, the overall estimated proportion of the population reporting to participate in charitable giving was 0.38 (95% CI [0.31, 0.46]), and the overall estimated proportion of the population reporting to participate in helping strangers was 0.56 (95% CI [0.49, 0.63]).

**Table 1:** Nationally representative descriptive statistics of the observed sample.

Characteristic	*N* = 202,898^1^
Age group	
18–24	27,007 (13%)
25–29	20,700 (10%)
30–39	40,256 (20%)
40–49	34,464 (17%)
50–59	31,793 (16%)
60–69	27,763 (14%)
70–79	16,776 (8.3%)
80 or older	4119 (2.0%)
(Missing)	20 (< 0.1%)
Gender	
Male	98,411 (49%)
Female	103,488 (51%)
Other	602 (0.3%)
(Missing)	397 (0.2%)
Marital status	
Married	107,354 (53%)
Separated	5195 (2.6%)
Divorced	11,654 (5.7%)
Widowed	9823 (4.8%)
Never	52,115 (26%)
Domestic Partner	14,931 (7.4%)
(Missing)	1826 (0.9%)
Employment	
Employed for an employer	78,815 (39%)
Self-employed	36,362 (18%)
Retired	29,303 (14%)
Student	10,726 (5.3%)
Homemaker	21,677 (11%)
Unemployed and looking for a job	16,790 (8.3%)
None of these/Other	8431 (4.2%)
(Missing)	793 (0.4%)
Religious service attendance	
> 1/week	26,537 (13%)
1/week	39,157 (19%)
1–3/month	19,749 (9.7%)
A few times a year	41,436 (20%)
Never	75,297 (37%)
(Missing)	722 (0.4%)
Education	
Up to 8 years	45,078 (22%)
9–15 years	115,097 (57%)
16 + years	42,578 (21%)
(Missing)	146 (< 0.1%)
Immigration	
Born in this country	190,998 (94%)
Born in another country	9791 (4.8%)
(Missing)	2110 (1.0%)
Country	
Argentina	6724 (3.3%)
Australia	3844 (1.9%)
Brazil	13,204 (6.5%)
Egypt	4729 (2.3%)
Germany	9506 (4.7%)
Hong Kong	3012 (1.5%)
India	12,765 (6.3%)
Indonesia	6992 (3.4%)
Israel	3669 (1.8%)
Japan	20,543 (10%)
Kenya	11,389 (5.6%)
Mexico	5776 (2.8%)
Nigeria	6827 (3.4%)
Philippines	5292 (2.6%)
Poland	10,389 (5.1%)
South Africa	2651 (1.3%)
Spain	6290 (3.1%)
Sweden	15,068 (7.4%)
Tanzania	9075 (4.5%)
Turkey	1473 (0.7%)
United Kingdom	5368 (2.6%)
United States	38,312 (19%)

^1^n (%).

Table 2 shows the ordered proportions of charitable giving and helping between countries. Indonesia had the highest proportion of individuals who reported charitable giving in the past month (0.79), followed by the UK (0.61), and Egypt (0.58); the 95% confidence intervals of Egypt and the UK overlapped. Conversely, Japan had the lowest proportion of people reporting charitable giving (0.10), followed by the Philippines (0.14), South Africa (0.19), Argentina (0.20), Poland (0.20), and Mexico (0.21), but notably the 95% confidence intervals of Mexico, Poland, Argentina, and South Africa overlapped.

**Table 2:** Ordered proportions of charitable giving and helping.

Charitable giving				Helping			
Country	Proportion	95% CI	SE	Country	Proportion	95% CI	SE
Indonesia	0.79	(0.77, 0.80)	0.009	Nigeria	0.83	(0.81, 0.84)	0.009
United Kingdom	0.61	(0.59, 0.63)	0.009	Egypt	0.73	(0.71, 0.74)	0.009
Egypt	0.58	(0.56, 0.60)	0.010	Brazil	0.69	(0.67, 0.70)	0.006
Israel	0.55	(0.52, 0.59)	0.019	Argentina	0.67	(0.65, 0.69)	0.008
Australia	0.53	(0.51, 0.55)	0.010	Kenya	0.66	(0.64, 0.68)	0.008
Sweden	0.52	(0.51, 0.53)	0.005	Hong Kong	0.65	(0.62, 0.67)	0.012
Hong Kong	0.51	(0.48, 0.53)	0.013	Israel	0.64	(0.61, 0.67)	0.017
United States	0.49	(0.48, 0.50)	0.006	Philippines	0.64	(0.62, 0.65)	0.009
Nigeria	0.48	(0.45, 0.50)	0.013	Mexico	0.63	(0.61, 0.65)	0.009
India	0.39	(0.37, 0.41)	0.010	Australia	0.62	(0.60, 0.64)	0.010
Germany	0.36	(0.35, 0.38)	0.006	Turkey	0.61	(0.58, 0.64)	0.016
Spain	0.36	(0.35, 0.38)	0.008	United States	0.59	(0.58, 0.60)	0.006
Brazil	0.31	(0.30, 0.32)	0.005	South Africa	0.58	(0.55, 0.61)	0.015
Turkey	0.31	(0.28, 0.34)	0.015	India	0.56	(0.54, 0.58)	0.010
Kenya	0.28	(0.26, 0.30)	0.009	United Kingdom	0.56	(0.54, 0.58)	0.010
Tanzania	0.27	(0.26, 0.29)	0.007	Spain	0.53	(0.51, 0.55)	0.009
Mexico	0.21	(0.19, 0.22)	0.007	Germany	0.51	(0.50, 0.53)	0.007
Poland	0.20	(0.19, 0.22)	0.010	Indonesia	0.50	(0.48, 0.52)	0.009
Argentina	0.20	(0.19, 0.22)	0.007	Sweden	0.44	(0.43, 0.45)	0.005
South Africa	0.19	(0.17, 0.22)	0.011	Tanzania	0.34	(0.32, 0.35)	0.008
Philippines	0.14	(0.13, 0.15)	0.007	Poland	0.26	(0.24, 0.28)	0.012
Japan	0.10	(0.09, 0.10)	0.002	Japan	0.11	(0.11, 0.12)	0.003

For helping strangers, Nigeria showed the highest proportion (0.83), followed by Egypt (0.73) and Brazil (0.69), although rates were similar in Argentina (0.67) and Kenya (0.66); their 95% confidence intervals overlapped with Brazil’s. The countries where the fewest report helping strangers were Japan (0.11), followed by Poland (0.26), Tanzania (0.34) and Sweden (0.44). None of their 95% confidence intervals overlapped.

**Fig. 1:**
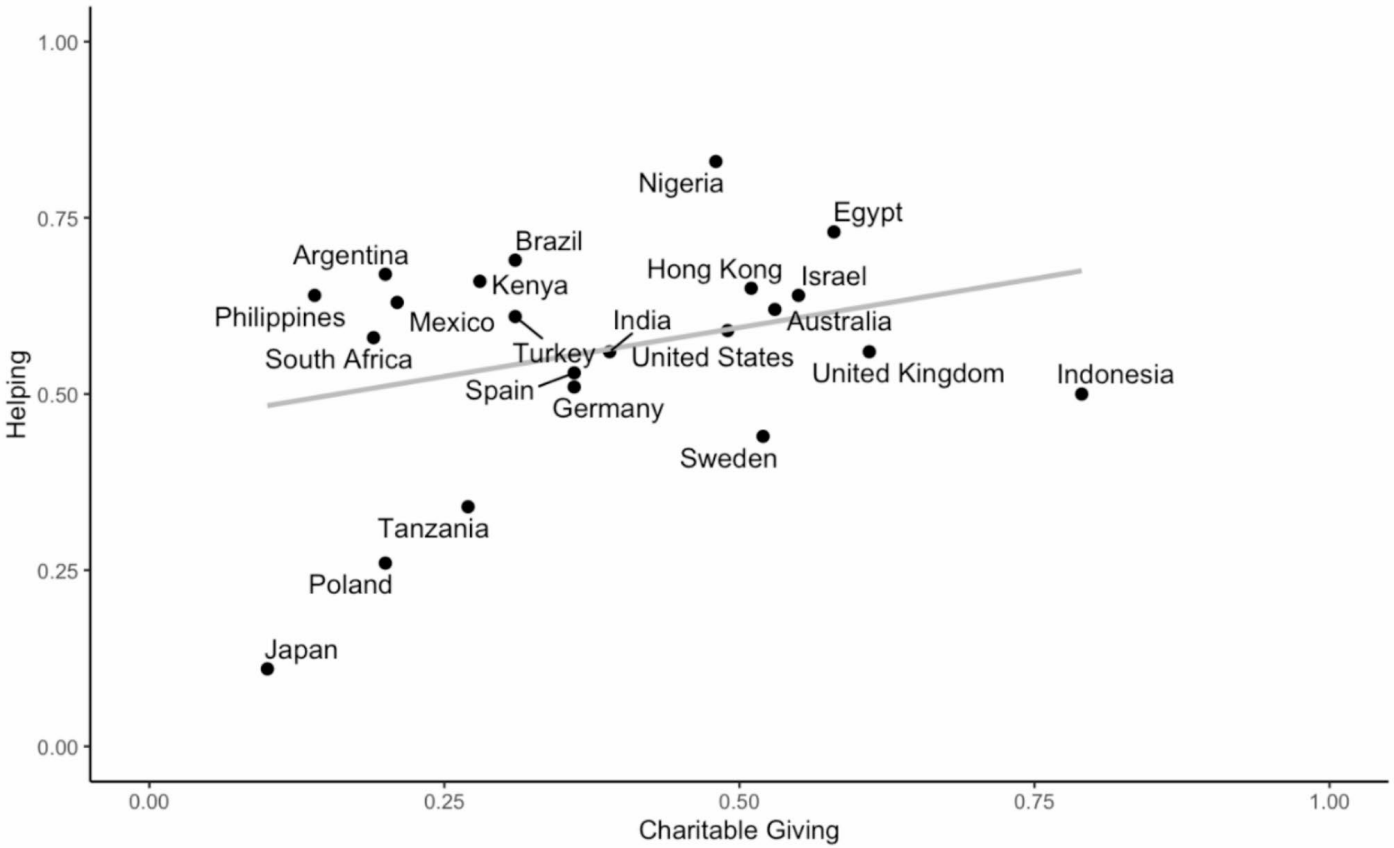
Scatterplot of correlations for charitable giving and helping at the country-level.

When we assessed both charitable giving and helping together, they were moderately correlated at the country-level (*r* = .31, *t*(20) = 1.46, *p* = .161; 95% CI: -0.13, 0.65). Figure 1 shows the correlations between charitable giving and helping at the country level.

Table 3 shows random meta-analytic effects of *charitable giving* across all demographic factors. The proportion of charitable giving increased with age: 28% of those 18–24 reported charitable giving (95% CI [0.22, 0.36]), as compared to 59% of those 80 or older (95% CI [0.32, 0.81]). Rates of charitable giving also differed based on marital status: 40% of married people (95% CI [0.31, 0.49]), 40% of widowed individuals (95% CI [0.31, 0.50]), and 37% of divorced people (95% CI [0.30, 0.45]) reported charitable giving, while only 29% of those with a domestic partner (95% CI [0.23, 0.36]) and 31% of those who were single but never married (95% CI [0.25, 0.39]) reported charitable giving. Rates of charitable giving also differed based on employment status: 41% of retired people (95% CI [0.32, 0.50]), 40% of self-employed people (95% CI [0.32, 0.49]), and 38% of employed people (95% CI [0.31, 0.47]) reported charitable giving, while only 28% of students (95% CI [0.21, 0.36]), 26% of the unemployed (95% CI [0.20, 0.33]), and 29% of those who selected ‘other’ as their employment category reported charitable giving (95% CI [0.22, 0.37]). Those with more education (e.g., 46% for those with 16 + years of education (95% CI [0.38, 0.54]) gave to charities more than those with less education (e.g., 33% for those with up to 8 years of education (95% CI [0.25, 0.42]). More religious service attendance was associated with more charitable giving: 29% of those who never attended religious services gave (95% CI [0.22, 0.38]), whereas 51% of those who attended religious services more than once per week gave (95% CI [0.40, 0.62]). Neither gender nor immigration status were associated with rates of charitable giving in the meta-analysis across countries.

**Table 3:** Random effects meta-analysis of charitable giving proportions by demographic category.

Variable	Category	Proportion	95% CI of proportion	SE analogue (CI Width/4)	Prediction Interval				
					LL	UL	τ	I^2	Global *p*-value
Age group									< 0.001**
	18–24	0.28	(0.22,0.36)	0.04	0.08	0.72	0.17	96.2	
	25–29	0.36	(0.28,0.44)	0.04	0.09	0.79	0.20	96.6	
	30–39	0.36	(0.28,0.44)	0.04	0.08	0.78	0.19	96.5	
	40–49	0.37	(0.28,0.46)	0.04	0.07	0.80	0.21	97.0	
	50–59	0.37	(0.29,0.46)	0.04	0.07	0.79	0.21	97.0	
	60–69	0.38	(0.30,0.47)	0.04	0.10	0.81	0.21	96.8	
	70–79	0.40	(0.31,0.49)	0.04	0.15	0.74	0.21	96.8	
	80 or older	0.59	(0.32,0.81)	0.12	0.05	1.00	0.64	99.6	
Gender									< 0.001**
	Male	0.38	(0.30,0.46)	0.04	0.10	0.76	0.19	96.3	
	Female	0.35	(0.27,0.44)	0.04	0.09	0.80	0.20	96.8	
	Other	0.08	(0.01,0.42)	0.10	0.00	1.00	0.34	99.8	
Marital status									< 0.001**
	Married	0.40	(0.31,0.49)	0.04	0.10	0.80	0.21	96.8	
	Separated	0.33	(0.27,0.40)	0.03	0.11	0.64	0.16	95.2	
	Divorced	0.37	(0.30,0.45)	0.04	0.09	0.79	0.18	96.1	
	Widowed	0.40	(0.31,0.50)	0.05	0.13	0.81	0.23	97.3	
	Domestic partner	0.29	(0.23,0.36)	0.03	0.13	0.60	0.14	95.0	
	Single, never married	0.31	(0.25,0.39)	0.04	0.08	0.71	0.17	95.9	
Employment status									< 0.001**
	Employed for an employer	0.38	(0.31,0.47)	0.04	0.09	0.75	0.19	96.4	
	Self-employed	0.40	(0.32,0.49)	0.04	0.15	0.81	0.19	96.3	
	Retired	0.41	(0.32,0.50)	0.04	0.13	0.79	0.21	96.7	
	Student	0.28	(0.21,0.36)	0.04	0.06	0.71	0.18	96.7	
	Homemaker	0.33	(0.25,0.43)	0.05	0.10	0.81	0.21	97.3	
	Unemployed and looking for a job	0.26	(0.20,0.33)	0.03	0.06	0.70	0.15	96.0	
	None of these/other	0.29	(0.22,0.37)	0.04	0.06	0.74	0.18	96.7	
Education									< 0.001**
	Up to 8 years	0.33	(0.25,0.42)	0.04	0.10	0.79	0.20	96.8	
	9–15 years	0.35	(0.28,0.44)	0.04	0.08	0.76	0.20	96.6	
	16 + years	0.46	(0.38,0.54)	0.04	0.15	0.82	0.19	96.2	
Religious service attendance									< 0.001**
	> 1/week	0.51	(0.40,0.62)	0.05	0.20	0.85	0.26	97.7	
	1/week	0.48	(0.38,0.58)	0.05	0.15	0.80	0.24	97.5	
	1–3/month	0.41	(0.32,0.51)	0.05	0.13	0.77	0.22	97.2	
	A few times a year	0.36	(0.28,0.44)	0.04	0.11	0.74	0.19	96.6	
	Never	0.29	(0.22,0.38)	0.04	0.06	0.73	0.19	97.0	
Immigration status									< 0.001**
	Born in this country	0.37	(0.29,0.45)	0.04	0.10	0.78	0.20	96.6	
	Born in another country	0.32	(0.19,0.50)	0.08	0.00	0.89	0.38	99.2	

*p < .05; **p < .007 (Bonferroni corrected threshold).

*Proportion*: Estimated overall proportion in the category.

*95% CI of Proportion*: The 95% CI for the estimated overall proportion of people who reported charitable giving for each demographic category.

*SE Analogue (CI Width/4)*: Standard error for the estimated overall proportion for each demographic group.

*Prediction interval*: Reflects how the country-specific proportion vary. LL: Lower limit of the 95% prediction interval. UP: Upper limit of the 95% prediction interval.

τ (*tau; h*eterogeneity): Measures the standard deviation of the distribution of proportions across countries. It is an estimate of how much the proportion in that demographic category varies across countries.

*I^2*: Estimates how much of the variability in proportions is due to heterogeneity across countries versus sampling variability. Given that the sample sizes of this study are large, the I^2 is high.

*Global p-value*: Tests the null hypothesis that the demographic category does not matter in any of the 22 countries.

Table 4 shows random meta-analytic effects of *helping* across all demographic factors. The proportion of helping appears to decrease with age: 60% of those aged 18–24 reported helping a stranger in the past month (0.95% CI [0.53, 0.66]), as compared to 48% of those 80 or older (0.95% CI [0.29, 0.67]). Rates of helping also differed based on employment status. Among the most likely to report helping strangers were those who were self-employed (63%, 95% CI [0.55, 0.70]), employed (0.59, 95% CI [0.51, 0.67]), or students (0.58, 95% CI [0.51, 0.65]). Among the least likely to report helping strangers were those who were homemakers (0.51, 95% CI [0.43, 0.59]) or retired (0.48, 95% CI [0.40, 0.57]). Those with more education (e.g., 62% for those with 16 + years of education (95% CI [0.55, 0.70]) reported helping more than those with less education (e.g., 51% for those with up to 8 years of education (95% CI [0.43, 0.60]). More frequent religious service attendance was also associated with more helping: 51% of those who never attended religious services reported helping strangers (95% CI [0.44, 0.59]), whereas 64% of those who attended religious services more than once per week did (95% CI [0.57, 0.71]). Rates of helping did not differ across categories of marital status (though widowed individuals may have had a lower proportion of helping than other groups), gender, or immigration status.

**Table 4:** Random effects meta-analysis of helping proportions by demographic category.

Variable	Category	Proportion	95% CI of proportion	SE analogue (CI Width/4)	Prediction interval				
					LL	UL	τ	I^2	Global *p*-value
Age group									< 0.001**
	18–24	0.60	(0.53, 0.66)	0.03	0.19	0.75	0.16	94.9	
	25–29	0.59	(0.52, 0.67)	0.04	0.13	0.84	0.18	95.8	
	30–39	0.59	(0.52, 0.67)	0.04	0.14	0.84	0.18	95.6	
	40–49	0.58	(0.50, 0.65)	0.04	0.13	0.83	0.18	95.6	
	50–59	0.57	(0.48, 0.66)	0.04	0.11	0.88	0.21	96.7	
	60–69	0.51	(0.43, 0.60)	0.04	0.10	0.85	0.20	96.4	
	70–79	0.45	(0.37, 0.54)	0.04	0.10	0.83	0.20	96.4	
	80 or older	0.48	(0.29, 0.67)	0.09	0.09	1.00	0.47	99.3	
Gender									< 0.001**
	Male	0.58	(0.50, 0.65)	0.04	0.12	0.84	0.18	95.9	
	Female	0.54	(0.46, 0.61)	0.04	0.12	0.80	0.18	95.6	
	Other	0.56	(0.18, 0.88)	0.17	0.00	1.00	0.92	99.8	
Marital status									< 0.001**
	Married	0.55	(0.47, 0.63)	0.04	0.11	0.84	0.19	96.2	
	Separated	0.57	(0.48, 0.65)	0.04	0.16	0.83	0.21	96.8	
	Divorced	0.56	(0.48, 0.64)	0.04	0.13	0.80	0.19	96.1	
	Widowed	0.49	(0.41, 0.57)	0.04	0.12	0.84	0.19	96.1	
	Domestic partner	0.54	(0.47, 0.61)	0.04	0.18	0.70	0.16	94.7	
	Single, never married	0.57	(0.50, 0.64)	0.04	0.13	0.79	0.17	95.1	
Employment status									< 0.001**
	Employed for an employer	0.59	(0.51, 0.67)	0.04	0.13	0.85	0.18	95.9	
	Self-employed	0.63	(0.55, 0.70)	0.04	0.16	0.84	0.17	95.7	
	Retired	0.48	(0.40, 0.57)	0.04	0.08	0.87	0.21	96.7	
	Student	0.58	(0.51, 0.65)	0.04	0.16	0.73	0.16	95.0	
	Homemaker	0.51	(0.43, 0.59)	0.04	0.10	0.80	0.19	95.9	
	Unemployed and looking for a job	0.55	(0.46, 0.63)	0.04	0.09	0.78	0.20	96.4	
	None of these/other	0.52	(0.45, 0.60)	0.04	0.10	0.78	0.18	95.8	
Education									< 0.001**
	Up to 8 years	0.51	(0.43, 0.60)	0.04	0.13	0.82	0.20	96.3	
	9–15 years	0.56	(0.47, 0.63)	0.04	0.10	0.82	0.19	96.0	
	16 + years	0.62	(0.55, 0.70)	0.04	0.18	0.90	0.17	95.8	
Religious service attendance									< 0.001**
	> 1/week	0.64	(0.57, 0.71)	0.03	0.26	0.88	0.16	95.1	
	1/week	0.60	(0.53, 0.67)	0.03	0.19	0.80	0.16	95.0	
	1–3/month	0.60	(0.53, 0.67)	0.04	0.23	0.81	0.16	95.1	
	A few times a year	0.57	(0.49, 0.64)	0.04	0.16	0.81	0.17	95.0	
	Never	0.51	(0.44, 0.59)	0.04	0.10	0.81	0.18	95.5	
Immigration status									< 0.001**
	Born in this country	0.56	(0.48, 0.63)	0.04	0.12	0.82	0.18	95.8	
	Born in another country	0.59	(0.51, 0.65)	0.04	0.23	0.90	0.17	95.1	

Note: *p < .05; **p < .007 (Bonferroni corrected threshold).

*Proportion*: Estimated overall proportion in the category.

*95% CI of Proportion*: The 95% CI for the estimated overall proportion of people who reported helping for each demographic category.

*SE Analogue (CI Width/4)*: Standard error for the estimated overall proportion for each demographic group.

*Prediction interval*: Reflects how the country-specific proportion vary. LL: Lower limit of the 95% prediction interval. UP: Upper limit of the 95% prediction interval.

τ (*tau; h*eterogeneity): Measures the standard deviation of the distribution of proportions across countries. It is an estimate of how much the proportion in that demographic category varies across countries.

*I^2*: Estimates how much of the variability in proportions is due to heterogeneity across countries versus sampling variability. Given that the sample sizes of this study are large, the I^2 is high.

*Global p-value*: Tests the null hypothesis that the demographic category does not matter in any of the 22 countries.

Tables 3 and 4 do not show proportions between either religious groups or racial and ethnic groups because these groups are different in different countries. Since Tables 3 and 4 show results pooled across all countries, we only show the demographic categories that had the same categories across countries. Tables S1b-S22b in the Supplementary Material show the proportions of people who reported charitable giving and helping across religious groups and racial and ethnic groups. The “tau” estimate shows how much the proportion of people who reported charitable giving or helping in that demographic category varied on average across countries—i.e., the standard deviation of the proportions across countries. In our study, the “tau” estimate suggested some variation in the proportions across countries, with τ ranging between 0.14 and 0.92 (Tables 3 and 4). The variation was particularly large among the ‘other’ gender category for helping strangers (τ = 0.92) and the 80 or older age group for charitable giving (τ = 0.64), which is likely due to the large uncertainty in a few of the country estimates. The estimated prediction intervals were also wide across sociodemographic categories, adding further evidence of heterogeneity across countries.

Supplementary Tables S1b-S22b show the proportions of charitable giving and helping for each demographic category for each country. Supplementary Figures S1-S115 provide forest plots for demographic factors by country for charitable giving and helping. In most countries charitable giving increased with age, although there were some exceptions (e.g., in Kenya, giving decreased with age). Some countries showed decreases in helping with age, though several showed no significant age differences or other age-related patterns (e.g., Poland, Mexico). For gender, results were mixed; in some countries males reported more charitable giving (e.g., Germany, Tanzania, Spain), while females reported more charitable giving in a few countries (e.g., Australia, Sweden), and elsewhere no differences were evident. Results for marital status and employment status were mixed. Regarding employment status, being employed or self-employed was associated with greater charitable giving (e.g., Hong Kong) and helping (e.g., Argentina, Brazil, Israel, Poland). In several countries, being retired was associated with more charitable giving (e.g., Brazil, Poland, Sweden, US), and being a student was associated with more helping in several countries (e.g., Germany, Indonesia, Sweden).

For 19 countries, more education was associated with more charitable giving; for two (Hong Kong and India) there was no association; and for Israel less education was associated with more charitable giving. Similarly, in 17 countries, more education was associated with more helping (but not in Hong Kong, India, Israel, Spain, and the Philippines). In 17 countries, more frequent religious service attendance was associated with more charitable giving. However, in Egypt, there was no clear trend, and in Nigeria, Kenya, South Africa, and Turkey there was little evidence of an association. Similarly, in 15 countries, the frequency of religious service attendance was associated with more helping; in Poland there was no clear trend; and in six countries (Australia, Nigeria, Turkey, Egypt, Kenya, and South Africa) the association was not statistically significant. Findings for immigration status were mixed.

Supplementary Tables 23a and 23b show an alternative meta-analysis where each country’s results are weighted by their 2023 population size for charitable giving and helping, respectively. These results produced fairly similar results to our main meta-analysis results.

## Discussion

Using data from a diverse and international sample of 202,898 individuals across 22 countries, we examined the distributions of charitable giving and helping strangers across countries and demographic factors.

The proportions of participants reporting charitable giving varied between countries. The top three countries for charitable giving (Indonesia, the UK, Egypt) all reported rates of 58% or greater, while the bottom three countries (South Africa, Philippines, and Japan) had rates of lower than 20%. These findings are similar to prior research in 2022 (e.g., in which 82% of individuals in Indonesia donated money (79% in our study), 71% of individuals in the UK donated money (61% in the current study), and South Africa, Philippines, and Japan all report charitable giving rates below 25%)^34^.

In one case, Egypt, the data do not match previous estimates. In our survey 58% of Egyptians reported charitable giving. This is much higher than the percent of Egyptians reporting giving in any year of the World Giving Index (WGI 2010–2023, corresponding to the years 2009–2022)^62^. In the 2010 WGI, 19% of Egyptians reported charitable giving; in the 2023 WGI, 9% of Egyptians reported charitable giving^62^. As could be the case in other countries, perhaps due to the timing of the survey (e.g., some surveys being conducted during and after Ramadan when many Muslims may have given for religious reasons) and in relation to current events (e.g., our survey was taken after the 2023 WGI, but before the conflict between Israel and Gaza), estimates seem to be higher than expected.

In contrast to what might be expected, gross domestic product (GDP) per capita does not appear to have a clear zero-order correlation with charitable giving. When evaluating the correlation between GDP per capita in 2023 in USD (using World Bank Data) and our proportions of GDP at the country-level, we found little evidence of correlation (*r* = .12, *t*(20) = 0.56, *p* = .58; 95% CI: -0.31, 0.52); wealthy and poor countries were at the top, middle, and bottom of the ranking in percentages for this prosocial behavior (see Table 5)^63^. For example, Japan, which is relatively wealthy (ranked 3rd for GDP in our study) had the lowest proportion of charitable giving (0.10, ranked 22nd), while Indonesia, which is relatively more poor (ranked 10th for GDP in our study) had the highest proportion of charitable giving (0.79).

**Table 5:** Charitable giving and GDP rankings.

Country	Charitable giving proportion	Charitable giving ranking	GDP (in USD in 2023)	GDP ranking in our study
Indonesia	0.79	1	1.37E + 12	10
United Kingdom	0.61	2	3.38E + 12	5
Egypt	0.58	3	3.96E + 11	17
Israel	0.55	4	5.14E + 11	15
Australia	0.53	5	1.73E + 12	8
Sweden	0.52	6	5.85E + 11	14
Hong Kong	0.51	7	3.81E + 11	18
United States	0.49	8	2.77E + 13	1
Nigeria	0.48	9	3.64E + 11	20
India	0.39	10	3.57E + 12	4
Germany	0.36	12	4.53E + 12	2
Spain	0.36	11	1.62E + 12	9
Brazil	0.31	13	2.17E + 12	6
Turkey	0.31	13	1.12E + 12	11
Kenya	0.28	15	1.08E + 11	21
Tanzania	0.27	16	7.91E + 10	22
Mexico	0.21	17	1.79E + 12	7
Poland	0.20	18	8.09E + 11	12
Argentina	0.20	18	6.46E + 11	13
South Africa	0.19	20	3.81E + 11	19
Philippines	0.14	21	4.37E + 11	16
Japan	0.10	22	4.20E + 12	3

Several factors may increase charitable giving, including, but not limited to: cultural values, large religious populations, national policies promoting charitable donations (e.g., the UK government’s Gift Aid program that increases the value of donations at no additional cost to the donor), the prevalence of non-governmental organizations (NGOs; more NGOs may increase public awareness of causes and promote charitable giving through campaigns and outreach), high income per capita, and lack of welfare states (welfare states that provide strong social safety nets but may discourage charitable giving).

Social origins theory predicts that liberal democracies would have the highest rates of charitable giving, followed by corporatist and social-democratic countries^64,65^. This is potentially the case because in liberal democracies, (a) the state provides fewer services and people believe that providing for basic needs is the joint responsibility of the state and charitable organizations, and (b) the state does little to regulate or control nonprofits; thus, many nonprofit organizations compete in soliciting donations^64,65^. Conversely, in corporatist and social-democratic countries, the government takes a larger responsibility for social welfare, potentially decreasing donations^64,65^. We see some evidence of this, where the UK, Israel, Australia, and the US, all of which may be considered liberal democracies, are ranked within the top eight countries for charitable giving. Conversely, there are exceptions. For example, Indonesia, Egypt, and Sweden are all also ranked within the top eight countries, despite being social democratic (in the case of Sweden) or Statist (in the case of Egypt and Indonesia). Anheier and Salamon also discuss their theory in their work^68,69^. Interestingly, all these countries are either historically Protestant or were colonized by Protestant colonizers (the British or the Dutch) and thus had high exposure to Protestant missions and religious competition^66,67^.

Our findings align with prior work that found some, but mixed empirical support for predictions about charitable giving behaviors taken from Salamon and Anheier’s social origins theory (as one example, previous work shows that people in liberal democracies may not be more likely to donate, but that they may donate larger amounts)^65^. Indeed, social origins theory also makes predictions about the types of donations made (i.e., to which types of organizations) and the amounts donated, which we cannot assess in the current study^64,65^. In our study, broadly, the highest-ranked countries (the top eight listed above) had mostly either strong religious charitable traditions or were liberal democracies, while the lowest had mostly statist traditions with strong state control or weak nonprofit sectors.

Beyond overall country-level variation, social origins theory also provides a framework for understanding how relationships between individual demographic factors and charitable giving may differ cross-nationally. For example, education is associated with charitable giving in many contexts, but its effect may be more pronounced in liberal democracies where philanthropy is culturally emphasized and tax incentives exist to encourage donations (e.g., in the US, where proportions of charitable giving were 0.23 for those with up to 8 years of education, 0.45 for those with 9–15 years of education, and 0.58 for those with 16 + years of education; Supplementary Table 22b). In contrast, in social-democratic countries with strong welfare states, higher education may not be as strongly linked to charitable giving because individuals may perceive less necessity for private donations, as needs are addressed through taxation and public welfare systems. Indeed, we see this in Sweden, where education is less clearly associated with charitable giving. In Sweden, proportions of charitable giving were 0.59 for those with up to 8 years of education, 0.49 for those with 9–15 years of education, and 0.60 for those with 16 + years of education; Supplementary Table 18b).

The proportion of participants who reported helping a stranger in the past month suggests that different dynamics are at play. Again, helping rates varied meaningfully between countries. The five countries who reported the highest rates of helping strangers (Nigeria, Egypt, Brazil, Argentina, Kenya) all reported rates of 66% or greater, while the five countries near the bottom of the distribution (Indonesia, Sweden, Tanzania, Poland, Japan) all reported rates of 50% or less. All countries in Central and South America reported rates over 60% (e.g., Brazil, Argentina, and Mexico). These findings mostly mirror prior research in which African and Central and South American countries made up 7 of the 10 top ranked countries for helping a stranger in 2022^34^. Many countries reporting higher rates of helping strangers have low GDPs per capita and low charitable giving, so, as one potential explanation, there could be a tradeoff where people in countries with fewer financial resources are more likely to help others directly and people in wealthier countries are more likely to give money rather than helping directly. Alternatively, cultural norms around informal versus institutionalized giving, differing definitions of what constitutes helping across contexts, and many other factors could potentially explain these differences.

However, the tradeoff between charitable giving and helping strangers is not universal. Some countries rank high on both (e.g., Israel, Hong Kong). Others rank low on both (e.g., Tanzania, Poland, and Japan). Japan ranked the lowest for both charitable giving (10%) and helping strangers (11%). Japan’s lower proportions of these behaviors^70^ may be explained by social and economic factors unique to Japan (e.g., lower visibility of charitable organizations or different social norms concerning public generosity such as potential low trust of charitable organizations) or less religiosity (the percent of Japanese who identify as non-religious (61%) or who never attend religious services (77%) is higher than any other country in our sample). Of course, the Japanese may engage in prosocial behaviors that are not measured by volunteering, charitable giving, or helping strangers (e.g., filial piety). Other types of prosocial behaviors could potentially be measured by surveying tourists or marginalized communities about how helpful others are, or by qualitative interviews to identify how Japanese citizens define prosocial behavior. The observed patterns in Japan may also be explained by broader religious or cultural patterns and thus might be observed in other nations, rather than being unique to Japan.

Some scholars argue that voluntarism and giving are higher in former British colonies because they often had more religious liberty, greater exposure to non-state-supported missionaries, and greater religious competition^66,68,71,72^. The pattern in our data seem to be consistent with such theoretical perspectives. However, our data do not allow us to test this theory rigorously or to rule out other possible explanations.

Our random effects meta-analytic proportions suggest some demographic factors have contrasting impacts on charitable giving and helping. For example, age seems to have opposite associations with charitable giving and helping strangers. Charitable giving seems to increase with age, potentially due to increased financial stability, more disposable income, and greater social responsibility. In contrast, helping appears to decline with age: primarily for the older age groups (60–80 and above), perhaps because of age-related physical and/or cognitive health problems that impede helping strangers or reduce contact with strangers^11^. Although age is generally associated with less helping of strangers, this is not always the case. In some countries (e.g., Kenya, Poland, Mexico) helping does not seem to decrease with age. This may be because of extended families supporting older adults so they are not isolated from broader society (e.g., not in retirement homes).

However, some factors have similar associations with both charitable giving and helping. Both education and religious service attendance were associated with more charitable giving and more helping, perhaps because of: greater awareness of social issues, more prosocial values, greater income, more solicitation,; community involvement, and religious teachings, amongst other reasons^10,20^. Analyses in individual countries suggests that typically education is associated with both increased helping and charitable giving, although not in Hong Kong, India, Israel, and Spain.

Likewise, in most countries, religious service attendance was associated with more charitable giving and helping, with a few exceptions (e.g., Nigeria, Turkey, Egypt, Kenya, Australia, South Africa, and Poland). In several of these countries, religious service attendance was quite high (e.g., in Nigeria only 4.8% of the population attended religious services less than 1–3 times a month). It is possible that the religious culture may pervade much of the society, including those who are less tied to religious institutions.

Although some countries showed higher rates of giving for males than females, there were others where females had a higher proportion of charitable giving than males (e.g., Australia, Sweden). These ‘individualistic’ countries may provide females with more opportunities for employment, broader social networks, and greater community involvement. Differences between individual countries highlight the complexity of charitable giving and helping patterns and how they may be influenced by a multitude of cultural and social factors.

Our study has several limitations. First, item translation between languages may lead to different interpretations of our charitable giving and helping items. Charitable giving and helping are broad constructs, and participant interpretations may vary depending on cultural norms, personal experiences, and societal expectations (i.e., what constitutes ‘helping’ a stranger or ‘charity’ may differ across national contexts). Words may have different connotations in different languages (despite dedicated efforts to minimize this possibility). Because the meaning and practice of ‘charitable giving’ and ‘helping’ may vary across cultural and institutional contexts, future research should explore qualitative approaches (e.g., to understand how people interpret ‘helping’ a stranger), additional survey items (e.g., donating to religious organizations versus secular nonprofits versus other types of organizations), or other methodologies to capture further nuance in our understanding of these behaviors. Further, prosocial behaviors can take many forms, and charitable giving and helping are only two forms of prosocial engagement. In another manuscript using parallel methods, we have evaluated demographic variation in volunteering^73^. Future work may benefit from evaluating other forms of prosociality (e.g., empathy) which were not assessed in the GFS. Second, these are descriptive analyses and should not be interpreted causally (e.g., while employment may increase one’s likelihood of helping strangers, it is also possible that helping increases one’s likelihood of employment). The demographic descriptive statistics simply inform us of the proportion of people who helped in each demographic category. Third, these descriptive statistics capture patterns at a certain point in time – rates of charitable giving and helping in countries and across demographic groups may change over time and our findings may be influenced by seasonal effects (e.g., increased charitable giving during certain months of the year). For instance, the timing of surveys may influence charitable giving “in the past month” (e.g., Muslims often give the Zakat during Eid-al Fitr each year, and in the US, people give more in December, around the Christmas holidays, and before the tax year ends). We believe the GFS item asks about prosocial behaviors in the past month to maintain comparability across surveys and minimize concerns about recall bias over longer periods of time. Fourth, our assessment of charitable giving and helping was binary, and thus does not capture important nuance in prosocial engagement (e.g., what kind of helping behaviors occur, the amounts and targets of charitable giving, the type of organization money was given to [e.g., religious versus secular], etc.). Future studies may benefit from evaluating such nuances. For all the reasons above, we must be careful about interpreting specific rankings; confidence intervals moreover sometimes overlap. Our study also has several strengths, including comparison of a large number of demographic variables across many countries, consistency in operationalization of variables, and nationally representative samples^40^. Indeed, while there are already several other important multi-country international datasets, such as the Gallup World Poll and World Values Survey, the GFS has the advantage of a much wider breadth of well-being constructs, and also a longitudinal panel data structure starting in Wave 2, all of which will become more evident in future waves^41^.

In this study, we examined the distributions of charitable giving and helping across countries and demographic groups, using data from a diverse and international sample of 202,898 individuals across 22 countries. As we continue to identify the substantial contributions of charitable giving and helping to the well-being of individuals and societies, it is important to understand how participation in these activities differs across nations and sociodemographic groups. These findings may have significant implications for non-profit organizations and policymakers. Understanding that prosocial behaviors vary significantly by country can aid in tailoring outreach and engagement strategies and should serve as a caution to policymakers to consider their specific context and avoid a one-size-fits-all approach to efforts to encourage prosociality. Private, not-for-profit organizations may benefit from first identifying cultural norms and expectations in different regions, and then adapting communication and fundraising strategies that align with these. For example, leveraging support from community networks, such as faith-based groups, could contribute to increased charitable giving in some regions such as Kenya, where charitable giving may be underdeveloped, but religious service participation may be high. For policymakers, these insights could guide the development of social programs and incentives that encourage forms of prosocial behavior that are less prevalent yet beneficial to societal cohesion and welfare. For instance, governments might consider tax incentives for charitable giving, integrate encouragement of helping into educational curricula, identify areas where social infrastructure (e.g., community centers) may be lacking, or invest in programs that provide opportunities for prosocial engagement, amongst other potential steps that could be taken. In Japan for example, where charitable giving rates remain relatively low even among more educated and employed individuals, governments might consider tax incentives to encourage greater participation in philanthropy. In other countries where strong welfare states may reduce the perceived need for charitable donations (e.g., Poland), governments could consider integrating encouragement of helping into educational curricula to promote greater prosocial engagement across the educational spectrum. Better understanding where charitable giving and helping are (or are not) common may enable organizations to enhance prosociality across different cultural contexts around the world. With further research, these findings may inform policy making and interventions to enhance global prosociality in ways that are both culturally relevant and contextually appropriate.

## Electronic supplementary material

Below is the link to the electronic supplementary material.

Supplementary Material 1

## Data Availability

Data for Wave 1 of the Global Flourishing Study is available through the Center for Open Science upon submission of a pre-registration (https://doi.org/10.17605/OSF.IO/3JTZ8). Please see https://www.cos.io/gfs-access-data for more information about data access. All analyses were pre-registered with COS prior to data access (https://doi.org/10.17605/OSF.IO/E9URJ); all code to reproduce analyses are openly available in an online repository (https://doi.org/10.17605/OSF.IO/VBYPE).
